# CASE STUDY - TREATMENT OF AMIODARONE-INDUCED THYROID DYSFUNCTION IN LONG TERM CARE PATIENT WITH DEMENTIA

**DOI:** 10.1093/geroni/igad104.3212

**Published:** 2023-12-21

**Authors:** John Chen, Ling Ling Cheng

**Affiliations:** Kaiser Permanente Med Group/Southern California, Baldwin Park, California, United States; Kaiser Permanente Med Group/Southern California, Harbor City, California, United States

## Abstract

Pharmacodynamic and pharmacokinetic changes in geriatric population warrant a stepwise, patient-focused and interdisciplinary approach for treatment with potent, class III anti-arrhythmic Amiodarone by reviewing 1) benefits of cardiac tachyarrhythmia treatment, 2) risks of adverse drug events (ADE) i.e. pulmonary, hepatic and thyroid dysfunctions, 3) alternative treatment option(s) with consultation1. 81 years old African American female with history of stroke with vascular dementia, non-ST-elevation myocardial infarction (NSTEMI) and Atrial Fibrillation residing in long term care (LTC) was transferred via 911 for intractable supraventricular tachycardia (SVT) stabilized with Amiodarone treatment. The peak level of Thyroid Stimulation Hormone (TSH) 36.67 (reference range 0.35-4.0) reflexed the initiation of Levothyroxine for ADE Amiodarone-induced hypothyroidism, albeit thyrotoxicosis can also result2. Countering the prescribing cascade and polypharmacy, gradual uptitration of Levothyroxine and weaning of Amiodarone resulted in the nadir level of TSH 8.19 (reference range 0.35-4.0)2. Virtual consultation with Cardiology and Endocrinology Teams provided alternative treatment options with recommendations 1) taper off Amiodarone, 2) substitute with beta-blocker Bisoprolol for heart rate control and 3) continue Levothyroxine trending thyroid function tests. Further interdisciplinary research in vulnerable, older patients in LTC settings is imperative to align the risks, benefits, and alternative treatment option(s) of acute and chronic disease with fundamental geriatric principles, including continuous medication reconciliation, shared decision-making, and life care planning. 1 Basaria S, Cooper DS. Amiodarone and the thyroid. Am J Med. 2005;118(7):706-714. doi:10.1016/j.amjmed.2004.11.028 2 Danzi S, Klein I. Amiodarone-induced thyroid dysfunction. J Intensive Care Med. 2015;30(4):179-185. doi:10.1177/0885066613503278

